# Supplementary material for: Hot spot prediction in protein-protein interactions by an ensemble system
Source: BMC Syst Biol. 2018 Dec 31;12(Suppl 9):132. doi: 10.1186/s12918-018-0665-8 (PMC6311905; doi:10.1186/s12918-018-0665-8)
Supplement: Supplementary file 2 — All relevant MATLAB code. A simple Matlab implement of our predictor. (ZIP 19,742 kb) [file 12918_2018_665_MOESM2_ESM.zip › Additional file 2/Read ASA/ASA website/dbMPIKT/4BJ5A.html]

 NetSurfP - Protein Surface Accessibility and Secondary Structure Predictions

  

|  |  |  |
| --- | --- | --- |
|  |  | NetSurfP - Protein Surface Accessibility and Secondary Structure Predictions  Technical University of Denmark |

  


---

```
# For publication of results, please cite:
# A generic method for assignment of reliability scores applied to solvent accessibility predictions.
# Bent Petersen, Thomas Nordahl Petersen, Pernille Andersen, Morten Nielsen and Claus Lundegaard
# BMC Structural Biology 2009, 9:51 doi:10.1186/1472-6807-9-51
#
# Column 1: Class assignment - B for buried or E for Exposed - Threshold: 25% exposure, but not based on RSA
# Column 2: Amino acid
# Column 3: Sequence name
# Column 4: Amino acid number
# Column 5: Relative Surface Accessibility - RSA
# Column 6: Absolute Surface Accessibility
# Column 7: Z-fit score for RSA prediction
# Column 8: Probability for Alpha-Helix
# Column 9: Probability for Beta-strand
# Column 10: Probability for Coil
E G  4BJ5A                  1    0.879  69.209  -1.405   0.003   0.003   0.994
E G  4BJ5A                  2    0.658  51.792  -2.955   0.018   0.047   0.935
E G  4BJ5A                  3    0.544  42.789  -2.329   0.052   0.084   0.864
E R  4BJ5A                  4    0.556 127.301   0.441   0.064   0.216   0.721
E M  4BJ5A                  5    0.318  63.632  -0.233   0.066   0.296   0.638
E E  4BJ5A                  6    0.569  99.492   0.231   0.066   0.296   0.638
E H  4BJ5A                  7    0.410  74.634  -0.271   0.066   0.296   0.638
B V  4BJ5A                  8    0.249  38.333   0.013   0.066   0.296   0.638
E D  4BJ5A                  9    0.525  75.652  -0.752   0.064   0.216   0.721
E S  4BJ5A                 10    0.559  65.562  -1.120   0.052   0.084   0.864
E D  4BJ5A                 11    0.616  88.708  -1.201   0.113   0.087   0.800
B F  4BJ5A                 12    0.193  38.795  -0.162   0.191   0.086   0.723
E A  4BJ5A                 13    0.326  35.903   0.073   0.278   0.093   0.628
E P  4BJ5A                 14    0.382  54.263  -0.281   0.278   0.093   0.628
B I  4BJ5A                 15    0.161  29.803  -0.569   0.278   0.093   0.628
E R  4BJ5A                 16    0.432  98.836  -0.024   0.278   0.093   0.628
E R  4BJ5A                 17    0.504 115.485  -0.173   0.278   0.093   0.628
E S  4BJ5A                 18    0.461  54.041  -0.665   0.191   0.086   0.723
E K  4BJ5A                 19    0.526 108.136   0.201   0.118   0.150   0.732
E K  4BJ5A                 20    0.548 112.724   0.568   0.135   0.317   0.548
B V  4BJ5A                 21    0.226  34.675   0.117   0.135   0.317   0.548
B V  4BJ5A                 22    0.223  34.244  -0.380   0.125   0.227   0.648
E D  4BJ5A                 23    0.509  73.275  -0.413   0.191   0.086   0.723
E S  4BJ5A                 24    0.393  46.001  -0.800   0.430   0.016   0.555
E D  4BJ5A                 25    0.641  92.310  -0.862   0.622   0.015   0.363
E K  4BJ5A                 26    0.475  97.687   1.213   0.717   0.014   0.269
B I  4BJ5A                 27    0.081  14.929   0.193   0.751   0.050   0.199
B V  4BJ5A                 28    0.107  16.384   0.623   0.779   0.100   0.120
E K  4BJ5A                 29    0.532 109.474   1.361   0.779   0.100   0.120
B A  4BJ5A                 30    0.243  26.790   0.195   0.779   0.100   0.120
B I  4BJ5A                 31    0.075  13.875   0.167   0.660   0.049   0.291
E S  4BJ5A                 32    0.387  45.345  -0.162   0.561   0.047   0.393
E D  4BJ5A                 33    0.640  92.224  -0.087   0.622   0.015   0.363
E D  4BJ5A                 34    0.411  59.182  -0.132   0.622   0.015   0.363
B L  4BJ5A                 35    0.125  22.942  -0.176   0.622   0.015   0.363
E E  4BJ5A                 36    0.608 106.148   0.321   0.622   0.015   0.363
E Q  4BJ5A                 37    0.608 108.553   0.272   0.561   0.047   0.393
E K  4BJ5A                 38    0.528 108.548   0.536   0.268   0.043   0.689
E N  4BJ5A                 39    0.520  76.172  -0.402   0.191   0.086   0.723
B F  4BJ5A                 40    0.118  23.602   0.358   0.216   0.235   0.548
E T  4BJ5A                 41    0.363  50.320   0.486   0.268   0.505   0.227
B V  4BJ5A                 42    0.150  23.101   0.401   0.268   0.505   0.227
B L  4BJ5A                 43    0.135  24.664   0.033   0.268   0.505   0.227
E R  4BJ5A                 44    0.372  85.234   0.259   0.268   0.505   0.227
E K  4BJ5A                 45    0.459  94.375   0.127   0.169   0.612   0.219
B L  4BJ5A                 46    0.142  25.927   0.248   0.074   0.484   0.442
E N  4BJ5A                 47    0.461  67.490  -0.930   0.021   0.451   0.528
B L  4BJ5A                 48    0.181  33.196  -0.348   0.022   0.552   0.426
B V  4BJ5A                 49    0.188  28.972   0.703   0.023   0.655   0.322
E P  4BJ5A                 50    0.360  51.027  -0.390   0.022   0.552   0.426
B I  4BJ5A                 51    0.157  29.119  -0.343   0.021   0.451   0.528
E K  4BJ5A                 52    0.498 102.521   0.318   0.069   0.386   0.545
E K  4BJ5A                 53    0.546 112.333  -0.136   0.064   0.216   0.721
E S  4BJ5A                 54    0.516  60.510  -0.868   0.056   0.142   0.802
B V  4BJ5A                 55    0.258  39.716  -1.104   0.052   0.084   0.864
E S  4BJ5A                 56    0.638  74.809  -1.007   0.053   0.043   0.903
E S  4BJ5A                 57    0.529  61.964  -0.129   0.018   0.047   0.935
E P  4BJ5A                 58    0.406  57.668  -0.781   0.018   0.047   0.935
E K  4BJ5A                 59    0.588 121.054   0.261   0.019   0.141   0.840
E V  4BJ5A                 60    0.288  44.281  -0.096   0.021   0.279   0.699
B C  4BJ5A                 61    0.118  16.609  -1.555   0.021   0.279   0.699
E K  4BJ5A                 62    0.568 116.755   0.300   0.019   0.141   0.840
E P  4BJ5A                 63    0.416  59.030  -1.257   0.018   0.088   0.893
E S  4BJ5A                 64    0.548  64.226  -0.754   0.053   0.043   0.903
E P  4BJ5A                 65    0.467  66.225  -0.436   0.181   0.016   0.803
B V  4BJ5A                 66    0.181  27.804  -0.638   0.339   0.016   0.645
E K  4BJ5A                 67    0.487 100.155   0.584   0.430   0.016   0.555
E E  4BJ5A                 68    0.624 108.960   0.038   0.561   0.047   0.393
E R  4BJ5A                 69    0.359  82.211   0.117   0.660   0.049   0.291
B V  4BJ5A                 70    0.096  14.755   0.131   0.717   0.014   0.269
E D  4BJ5A                 71    0.386  55.680  -0.263   0.802   0.014   0.185
E H  4BJ5A                 72    0.388  70.541  -0.218   0.879   0.010   0.111
B V  4BJ5A                 73    0.174  26.805   0.192   0.938   0.007   0.055
B F  4BJ5A                 74    0.087  17.521   0.516   0.938   0.007   0.055
B Y  4BJ5A                 75    0.199  42.462   0.481   0.938   0.007   0.055
E Q  4BJ5A                 76    0.449  80.245   0.610   0.938   0.007   0.055
E K  4BJ5A                 77    0.401  82.403   0.425   0.879   0.010   0.111
B F  4BJ5A                 78    0.096  19.167  -0.238   0.879   0.010   0.111
E K  4BJ5A                 79    0.374  76.952   0.370   0.879   0.010   0.111
E S  4BJ5A                 80    0.518  60.663  -0.720   0.879   0.010   0.111
E M  4BJ5A                 81    0.276  55.148   0.552   0.802   0.014   0.185
B A  4BJ5A                 82    0.114  12.596  -0.792   0.879   0.010   0.111
B L  4BJ5A                 83    0.131  24.078  -0.756   0.879   0.010   0.111
E Q  4BJ5A                 84    0.514  91.747   0.492   0.879   0.010   0.111
E E  4BJ5A                 85    0.564  98.583   0.056   0.802   0.014   0.185
B L  4BJ5A                 86    0.157  28.747   0.342   0.622   0.015   0.363
E G  4BJ5A                 87    0.420  33.023  -1.076   0.257   0.016   0.727
E T  4BJ5A                 88    0.383  53.164  -0.742   0.113   0.043   0.844
E N  4BJ5A                 89    0.497  72.717  -0.893   0.052   0.084   0.864
B Y  4BJ5A                 90    0.195  41.629   0.091   0.069   0.386   0.545
B L  4BJ5A                 91    0.120  21.917  -0.116   0.023   0.655   0.322
B S  4BJ5A                 92    0.270  31.621  -0.587   0.021   0.756   0.223
B I  4BJ5A                 93    0.101  18.648  -0.180   0.023   0.655   0.322
E S  4BJ5A                 94    0.365  42.825  -0.880   0.022   0.552   0.426
B Y  4BJ5A                 95    0.222  47.399  -0.239   0.069   0.386   0.545
B V  4BJ5A                 96    0.130  20.027   0.422   0.118   0.150   0.732
E P  4BJ5A                 97    0.379  53.837  -0.567   0.268   0.043   0.689
E S  4BJ5A                 98    0.426  49.939  -0.769   0.522   0.016   0.462
B L  4BJ5A                 99    0.104  19.079  -0.270   0.782   0.003   0.216
E S  4BJ5A                100    0.317  37.106  -0.412   0.858   0.002   0.139
E K  4BJ5A                101    0.535 110.111   1.097   0.923   0.002   0.076
B F  4BJ5A                102    0.181  36.387  -0.367   0.923   0.002   0.076
B L  4BJ5A                103    0.091  16.717   0.165   0.923   0.002   0.076
E S  4BJ5A                104    0.431  50.548  -0.004   0.858   0.002   0.139
E K  4BJ5A                105    0.582 119.615   0.877   0.782   0.003   0.216
E N  4BJ5A                106    0.335  48.985  -0.830   0.694   0.003   0.303
B L  4BJ5A                107    0.107  19.500  -0.506   0.694   0.003   0.303
E R  4BJ5A                108    0.517 118.416   0.586   0.717   0.014   0.269
E S  4BJ5A                109    0.417  48.861   0.133   0.717   0.014   0.269
B M  4BJ5A                110    0.153  30.515  -0.373   0.622   0.015   0.363
E K  4BJ5A                111    0.439  90.241   0.050   0.354   0.048   0.598
E N  4BJ5A                112    0.377  55.266   0.272   0.199   0.152   0.649
B C  4BJ5A                113    0.027   3.847   0.522   0.159   0.516   0.325
B I  4BJ5A                114    0.059  11.007   0.015   0.085   0.775   0.140
B V  4BJ5A                115    0.072  11.113   0.133   0.072   0.855   0.073
B F  4BJ5A                116    0.060  12.142   0.090   0.157   0.773   0.069
B F  4BJ5A                117    0.067  13.487  -0.325   0.169   0.612   0.219
E D  4BJ5A                118    0.332  47.856  -0.233   0.252   0.423   0.325
E K  4BJ5A                119    0.337  69.218   0.021   0.321   0.252   0.427
B V  4BJ5A                120    0.097  14.924   0.098   0.386   0.097   0.517
E E  4BJ5A                121    0.499  87.193   0.462   0.502   0.102   0.396
E H  4BJ5A                122    0.287  52.133   0.238   0.502   0.102   0.396
B I  4BJ5A                123    0.074  13.635  -0.113   0.605   0.105   0.290
E H  4BJ5A                124    0.297  54.079   0.012   0.605   0.105   0.290
E Q  4BJ5A                125    0.548  97.855  -0.004   0.561   0.047   0.393
B Y  4BJ5A                126    0.233  49.835  -0.192   0.561   0.047   0.393
B A  4BJ5A                127    0.243  26.746  -0.629   0.354   0.048   0.598
E G  4BJ5A                128    0.394  31.000  -1.204   0.354   0.048   0.598
B I  4BJ5A                129    0.132  24.439   0.010   0.455   0.046   0.498
E D  4BJ5A                130    0.459  66.070  -0.482   0.522   0.016   0.462
E R  4BJ5A                131    0.459 105.019   0.514   0.717   0.014   0.269
B A  4BJ5A                132    0.227  24.982  -0.564   0.751   0.050   0.199
B V  4BJ5A                133    0.136  20.980   0.536   0.751   0.050   0.199
E S  4BJ5A                134    0.459  53.795  -0.268   0.660   0.049   0.291
E E  4BJ5A                135    0.617 107.825   0.296   0.605   0.105   0.290
E T  4BJ5A                136    0.282  39.183  -0.108   0.428   0.171   0.402
B L  4BJ5A                137    0.098  18.017   0.757   0.125   0.227   0.648
E S  4BJ5A                138    0.421  49.353   0.491   0.069   0.386   0.545
B L  4BJ5A                139    0.143  26.128   0.321   0.079   0.592   0.329
B V  4BJ5A                140    0.129  19.797   0.246   0.021   0.756   0.223
E D  4BJ5A                141    0.330  47.495  -0.489   0.021   0.756   0.223
B I  4BJ5A                142    0.080  14.744   0.272   0.021   0.756   0.223
B N  4BJ5A                143    0.256  37.478  -0.244   0.018   0.846   0.136
B V  4BJ5A                144    0.065  10.037   0.563   0.011   0.918   0.071
B V  4BJ5A                145    0.070  10.744   0.236   0.011   0.918   0.071
B I  4BJ5A                146    0.072  13.302   0.134   0.006   0.962   0.032
B I  4BJ5A                147    0.073  13.524   0.237   0.011   0.918   0.071
E E  4BJ5A                148    0.288  50.401   0.087   0.085   0.775   0.140
B M  4BJ5A                149    0.115  23.032   0.069   0.148   0.418   0.435
E N  4BJ5A                150    0.316  46.321  -0.572   0.278   0.093   0.628
E D  4BJ5A                151    0.408  58.793  -0.824   0.561   0.047   0.393
E Y  4BJ5A                152    0.281  60.028   0.187   0.751   0.050   0.199
B L  4BJ5A                153    0.095  17.431  -0.266   0.831   0.044   0.125
B M  4BJ5A                154    0.195  38.959   0.529   0.831   0.044   0.125
E K  4BJ5A                155    0.519 106.676   0.617   0.802   0.014   0.185
E E  4BJ5A                156    0.531  92.783   0.539   0.622   0.015   0.363
B G  4BJ5A                157    0.318  25.011  -1.650   0.430   0.016   0.555
B I  4BJ5A                158    0.137  25.401  -0.461   0.339   0.016   0.645
E Q  4BJ5A                159    0.581 103.838   0.077   0.257   0.016   0.727
E S  4BJ5A                160    0.508  59.514  -0.888   0.339   0.016   0.645
E S  4BJ5A                161    0.562  65.925  -1.464   0.430   0.016   0.555
E K  4BJ5A                162    0.559 115.069   0.026   0.522   0.016   0.462
E S  4BJ5A                163    0.285  33.379  -0.438   0.717   0.014   0.269
E K  4BJ5A                164    0.551 113.382   1.132   0.879   0.010   0.111
E E  4BJ5A                165    0.582 101.658   1.383   0.879   0.010   0.111
B C  4BJ5A                166    0.057   8.017  -0.045   0.938   0.007   0.055
B I  4BJ5A                167    0.124  22.847  -0.099   0.938   0.007   0.055
E E  4BJ5A                168    0.624 108.978   0.703   0.879   0.010   0.111
E S  4BJ5A                169    0.437  51.205   0.019   0.802   0.014   0.185
B M  4BJ5A                170    0.222  44.342  -0.020   0.622   0.015   0.363
E G  4BJ5A                171    0.430  33.825  -1.478   0.339   0.016   0.645
E Q  4BJ5A                172    0.544  97.230  -0.307   0.191   0.086   0.723
E A  4BJ5A                173    0.343  37.810  -1.525   0.118   0.150   0.732
E S  4BJ5A                174    0.489  57.334  -1.294   0.066   0.296   0.638
B Y  4BJ5A                175    0.250  53.382  -0.540   0.069   0.386   0.545
E S  4BJ5A                176    0.543  63.640  -0.969   0.021   0.279   0.699
E G  4BJ5A                177    0.391  30.772  -1.815   0.022   0.359   0.619
E Q  4BJ5A                178    0.506  90.389  -0.329   0.021   0.451   0.528
B L  4BJ5A                179    0.182  33.251   0.150   0.022   0.552   0.426
E D  4BJ5A                180    0.480  69.096  -0.224   0.022   0.552   0.426
B F  4BJ5A                181    0.186  37.270   0.063   0.022   0.552   0.426
E E  4BJ5A                182    0.593 103.545   0.160   0.079   0.592   0.329
E A  4BJ5A                183    0.270  29.754  -0.736   0.069   0.386   0.545
E S  4BJ5A                184    0.549  64.296  -1.000   0.064   0.216   0.721
E E  4BJ5A                185    0.647 113.048  -0.359   0.056   0.142   0.802
E K  4BJ5A                186    0.533 109.679   0.180   0.018   0.047   0.935
E P  4BJ5A                187    0.337  47.792  -1.361   0.018   0.019   0.964
E S  4BJ5A                188    0.588  68.902  -1.646   0.058   0.017   0.925
E N  4BJ5A                189    0.575  84.136  -1.142   0.115   0.016   0.868
B H  4BJ5A                190    0.302  54.861  -0.694   0.257   0.016   0.727
B T  4BJ5A                191    0.287  39.862  -1.234   0.858   0.002   0.139
E S  4BJ5A                192    0.385  45.110  -0.832   0.923   0.002   0.076
E D  4BJ5A                193    0.299  43.028  -0.517   0.970   0.001   0.030
B L  4BJ5A                194    0.057  10.382   0.677   0.975   0.003   0.022
B M  4BJ5A                195    0.097  19.390   0.068   0.975   0.003   0.022
B M  4BJ5A                196    0.173  34.577   0.653   0.975   0.003   0.022
B M  4BJ5A                197    0.142  28.414   0.904   0.975   0.003   0.022
B V  4BJ5A                198    0.048   7.424  -0.425   0.938   0.007   0.055
B M  4BJ5A                199    0.109  21.871  -0.109   0.938   0.007   0.055
E R  4BJ5A                200    0.398  91.073   0.465   0.911   0.033   0.057
E K  4BJ5A                201    0.433  89.130   0.446   0.831   0.044   0.125
B I  4BJ5A                202    0.086  15.855  -0.021   0.430   0.016   0.555
E N  4BJ5A                203    0.523  76.626  -0.477   0.115   0.016   0.868
E N  4BJ5A                204    0.620  90.797  -0.442   0.058   0.017   0.925
E D  4BJ5A                205    0.606  87.296  -1.585   0.115   0.016   0.868
E E  4BJ5A                206    0.656 114.673  -0.538   0.113   0.043   0.844
E S  4BJ5A                207    0.404  47.325   0.049   0.056   0.142   0.802
B I  4BJ5A                208    0.110  20.350   0.444   0.066   0.296   0.638
E D  4BJ5A                209    0.406  58.548  -0.235   0.135   0.317   0.548
B H  4BJ5A                210    0.219  39.872   0.534   0.087   0.683   0.230
B I  4BJ5A                211    0.072  13.357   0.290   0.072   0.855   0.073
B V  4BJ5A                212    0.064   9.837   0.618   0.072   0.855   0.073
B Y  4BJ5A                213    0.140  29.875   0.271   0.072   0.855   0.073
B F  4BJ5A                214    0.086  17.280   0.750   0.085   0.775   0.140
E K  4BJ5A                215    0.330  67.922   0.817   0.087   0.683   0.230
B F  4BJ5A                216    0.104  20.953   0.092   0.159   0.516   0.325
E E  4BJ5A                217    0.558  97.552   0.723   0.148   0.418   0.435
E Q  4BJ5A                218    0.451  80.549   0.404   0.216   0.235   0.548
B L  4BJ5A                219    0.155  28.417  -0.094   0.199   0.152   0.649
E D  4BJ5A                220    0.517  74.543  -0.443   0.191   0.086   0.723
E K  4BJ5A                221    0.564 115.953   0.619   0.199   0.152   0.649
B L  4BJ5A                222    0.172  31.475  -0.116   0.199   0.152   0.649
E S  4BJ5A                223    0.486  57.018  -0.815   0.199   0.152   0.649
E T  4BJ5A                224    0.449  62.290  -0.650   0.199   0.152   0.649
E S  4BJ5A                225    0.445  52.189  -0.850   0.125   0.227   0.648
E T  4BJ5A                226    0.334  46.395  -0.102   0.135   0.317   0.548
B I  4BJ5A                227    0.222  41.070  -0.351   0.069   0.386   0.545
B I  4BJ5A                228    0.185  34.170  -0.498   0.022   0.359   0.619
E E  4BJ5A                229    0.497  86.756   0.215   0.020   0.205   0.775
E P  4BJ5A                230    0.286  40.555  -0.675   0.184   0.043   0.773
E S  4BJ5A                231    0.608  71.304  -1.055   0.339   0.016   0.645
E K  4BJ5A                232    0.460  94.540   0.865   0.622   0.015   0.363
B L  4BJ5A                233    0.092  16.864  -0.182   0.802   0.014   0.185
E T  4BJ5A                234    0.302  41.901  -0.093   0.802   0.014   0.185
E E  4BJ5A                235    0.520  90.861   0.514   0.802   0.014   0.185
B F  4BJ5A                236    0.090  18.083   0.036   0.802   0.014   0.185
B I  4BJ5A                237    0.114  21.127   0.456   0.831   0.044   0.125
E N  4BJ5A                238    0.434  63.596  -0.008   0.831   0.044   0.125
B V  4BJ5A                239    0.079  12.188  -0.004   0.831   0.044   0.125
B L  4BJ5A                240    0.092  16.809   0.561   0.779   0.100   0.120
E S  4BJ5A                241    0.334  39.203   0.127   0.725   0.163   0.112
B V  4BJ5A                242    0.146  22.471   0.026   0.725   0.163   0.112
B L  4BJ5A                243    0.086  15.802   0.124   0.649   0.163   0.188
E E  4BJ5A                244    0.496  86.616   0.127   0.538   0.173   0.289
E K  4BJ5A                245    0.589 121.116  -0.096   0.386   0.097   0.517
E S  4BJ5A                246    0.441  51.709  -1.328   0.113   0.043   0.844
E N  4BJ5A                247    0.579  84.736  -1.621   0.053   0.043   0.903
E N  4BJ5A                248    0.466  68.178  -0.428   0.019   0.141   0.840
B I  4BJ5A                249    0.091  16.743   0.203   0.004   0.616   0.381
B A  4BJ5A                250    0.120  13.191  -0.796   0.001   0.900   0.099
B F  4BJ5A                251    0.061  12.203   0.225   0.001   0.900   0.099
B K  4BJ5A                252    0.172  35.298   0.679   0.001   0.959   0.040
B V  4BJ5A                253    0.036   5.472  -0.141   0.001   0.959   0.040
B L  4BJ5A                254    0.047   8.624   0.610   0.001   0.959   0.040
B I  4BJ5A                255    0.047   8.639  -0.094   0.001   0.959   0.040
B Y  4BJ5A                256    0.116  24.853  -0.195   0.001   0.900   0.099
B S  4BJ5A                257    0.213  24.905  -1.065   0.004   0.514   0.481
E N  4BJ5A                258    0.492  72.073  -1.232   0.018   0.088   0.893
E N  4BJ5A                259    0.575  84.195  -1.070   0.019   0.141   0.840
B V  4BJ5A                260    0.156  24.039  -0.185   0.069   0.386   0.545
E S  4BJ5A                261    0.342  40.082  -0.549   0.159   0.516   0.325
B I  4BJ5A                262    0.089  16.391  -0.013   0.478   0.309   0.214
E S  4BJ5A                263    0.334  39.157  -0.515   0.701   0.107   0.192
E S  4BJ5A                264    0.420  49.177  -0.354   0.831   0.044   0.125
B L  4BJ5A                265    0.169  30.981  -0.138   0.831   0.044   0.125
B L  4BJ5A                266    0.104  19.006   0.023   0.831   0.044   0.125
E S  4BJ5A                267    0.387  45.356  -0.260   0.831   0.044   0.125
E T  4BJ5A                268    0.428  59.419  -0.473   0.802   0.014   0.185
E S  4BJ5A                269    0.349  40.915  -0.737   0.802   0.014   0.185
B L  4BJ5A                270    0.112  20.507  -0.411   0.802   0.014   0.185
E K  4BJ5A                271    0.449  92.318   0.878   0.831   0.044   0.125
E K  4BJ5A                272    0.600 123.317   0.509   0.751   0.050   0.199
E K  4BJ5A                273    0.506 104.084   0.312   0.660   0.049   0.291
B L  4BJ5A                274    0.130  23.821  -0.372   0.455   0.046   0.498
E N  4BJ5A                275    0.630  92.261  -0.346   0.191   0.086   0.723
E T  4BJ5A                276    0.364  50.501  -0.321   0.118   0.150   0.732
E K  4BJ5A                277    0.508 104.537   0.528   0.066   0.296   0.638
B Y  4BJ5A                278    0.166  35.538   0.876   0.079   0.592   0.329
B T  4BJ5A                279    0.242  33.538   0.062   0.023   0.655   0.322
B V  4BJ5A                280    0.145  22.256  -0.060   0.085   0.775   0.140
B F  4BJ5A                281    0.116  23.321  -0.163   0.085   0.775   0.140
E E  4BJ5A                282    0.428  74.719  -0.188   0.079   0.592   0.329
B M  4BJ5A                283    0.135  27.014   1.021   0.069   0.386   0.545
B P  4BJ5A                284    0.231  32.765  -0.508   0.135   0.317   0.548
B I  4BJ5A                285    0.134  24.716  -0.437   0.231   0.330   0.439
B L  4BJ5A                286    0.152  27.831   0.140   0.231   0.330   0.439
E T  4BJ5A                287    0.256  35.535   0.017   0.352   0.332   0.316
B C  4BJ5A                288    0.074  10.460  -0.276   0.538   0.173   0.289
E A  4BJ5A                289    0.353  38.934  -0.453   0.660   0.049   0.291
E Q  4BJ5A                290    0.515  91.908  -0.184   0.622   0.015   0.363
E E  4BJ5A                291    0.315  55.083  -0.259   0.802   0.014   0.185
E Q  4BJ5A                292    0.406  72.440   0.195   0.938   0.007   0.055
E E  4BJ5A                293    0.644 112.454   0.876   0.923   0.002   0.076
B Y  4BJ5A                294    0.212  45.390   0.528   0.975   0.003   0.022
B L  4BJ5A                295    0.058  10.565   0.323   0.938   0.007   0.055
E K  4BJ5A                296    0.465  95.630   1.409   0.938   0.007   0.055
E K  4BJ5A                297    0.488 100.402   0.994   0.879   0.010   0.111
B M  4BJ5A                298    0.125  25.013   0.390   0.831   0.044   0.125
B I  4BJ5A                299    0.064  11.877   0.286   0.701   0.107   0.192
E K  4BJ5A                300    0.486  99.888   1.419   0.478   0.309   0.214
B F  4BJ5A                301    0.150  30.065   0.459   0.268   0.505   0.227
E T  4BJ5A                302    0.271  37.574  -0.148   0.159   0.516   0.325
B F  4BJ5A                303    0.149  29.944  -0.145   0.148   0.418   0.435
E D  4BJ5A                304    0.570  82.180  -0.517   0.199   0.152   0.649
E S  4BJ5A                305    0.480  56.268  -0.954   0.181   0.016   0.803
B G  4BJ5A                306    0.331  26.089  -1.873   0.321   0.003   0.675
E S  4BJ5A                307    0.415  48.638  -0.271   0.622   0.015   0.363
E K  4BJ5A                308    0.533 109.556   1.158   0.751   0.050   0.199
B L  4BJ5A                309    0.131  23.968   0.384   0.751   0.050   0.199
B L  4BJ5A                310    0.115  21.057  -0.031   0.751   0.050   0.199
E Q  4BJ5A                311    0.567 101.195   0.164   0.751   0.050   0.199
E S  4BJ5A                312    0.444  51.990  -0.331   0.605   0.105   0.290
B Y  4BJ5A                313    0.183  39.000  -0.159   0.386   0.097   0.517
E N  4BJ5A                314    0.522  76.348  -0.957   0.113   0.087   0.800
B S  4BJ5A                315    0.292  34.176  -0.552   0.066   0.296   0.638
B L  4BJ5A                316    0.140  25.597  -0.173   0.021   0.756   0.223
B V  4BJ5A                317    0.086  13.264   0.038   0.011   0.918   0.071
B T  4BJ5A                318    0.239  33.094   0.196   0.011   0.918   0.071
B C  4BJ5A                319    0.042   5.939  -0.117   0.011   0.918   0.071
E Q  4BJ5A                320    0.333  59.527   0.218   0.011   0.918   0.071
B L  4BJ5A                321    0.098  17.980  -0.492   0.021   0.756   0.223
E N  4BJ5A                322    0.487  71.282  -0.186   0.022   0.359   0.619
E N  4BJ5A                323    0.557  81.516  -0.780   0.018   0.088   0.893
E K  4BJ5A                324    0.585 120.417  -0.253   0.056   0.142   0.802
E E  4BJ5A                325    0.653 114.166  -0.761   0.125   0.227   0.648
E S  4BJ5A                326    0.368  43.106  -0.503   0.191   0.086   0.723
B N  4BJ5A                327    0.272  39.850  -0.244   0.428   0.171   0.402
B L  4BJ5A                328    0.078  14.300   0.449   0.594   0.285   0.121
B A  4BJ5A                329    0.176  19.384  -0.109   0.614   0.324   0.062
B I  4BJ5A                330    0.084  15.540   0.545   0.685   0.273   0.043
B F  4BJ5A                331    0.041   8.269   0.186   0.731   0.235   0.034
B F  4BJ5A                332    0.117  23.402   0.227   0.807   0.137   0.056
E E  4BJ5A                333    0.392  68.482   0.425   0.870   0.077   0.053
B F  4BJ5A                334    0.098  19.709   0.169   0.831   0.044   0.125
B L  4BJ5A                335    0.112  20.580   0.258   0.751   0.050   0.199
E K  4BJ5A                336    0.477  98.119   0.342   0.660   0.049   0.291
B V  4BJ5A                337    0.237  36.396  -0.634   0.455   0.046   0.498
B F  4BJ5A                338    0.118  23.663   0.717   0.113   0.043   0.844
E P  4BJ5A                339    0.458  64.933  -0.902   0.058   0.017   0.925
B H  4BJ5A                340    0.315  57.262  -0.370   0.058   0.017   0.925
B P  4BJ5A                341    0.298  42.315  -0.850   0.268   0.043   0.689
B F  4BJ5A                342    0.125  25.148  -0.165   0.502   0.102   0.396
B T  4BJ5A                343    0.246  34.051  -0.186   0.649   0.163   0.188
B Y  4BJ5A                344    0.193  41.201   0.105   0.649   0.163   0.188
B L  4BJ5A                345    0.079  14.410  -0.367   0.725   0.163   0.112
B F  4BJ5A                346    0.095  19.107   0.315   0.649   0.163   0.188
E N  4BJ5A                347    0.371  54.329  -0.278   0.502   0.102   0.396
B A  4BJ5A                348    0.136  14.954  -0.081   0.502   0.102   0.396
B Y  4BJ5A                349    0.142  30.239  -0.107   0.578   0.229   0.194
B T  4BJ5A                350    0.172  23.829  -0.138   0.453   0.248   0.299
E E  4BJ5A                351    0.420  73.322   0.397   0.478   0.309   0.214
B I  4BJ5A                352    0.093  17.279   0.270   0.487   0.370   0.144
B I  4BJ5A                353    0.092  17.020   0.427   0.487   0.370   0.144
B V  4BJ5A                354    0.157  24.131  -0.278   0.391   0.466   0.143
E Q  4BJ5A                355    0.375  67.046  -0.082   0.352   0.332   0.316
E S  4BJ5A                356    0.296  34.750  -0.860   0.307   0.165   0.527
E R  4BJ5A                357    0.449 102.890   0.021   0.386   0.097   0.517
E T  4BJ5A                358    0.304  42.095   0.156   0.455   0.046   0.498
B F  4BJ5A                359    0.073  14.651   0.256   0.879   0.010   0.111
E D  4BJ5A                360    0.486  70.105   0.478   0.923   0.002   0.076
E E  4BJ5A                361    0.553  96.627   1.390   0.923   0.002   0.076
B L  4BJ5A                362    0.067  12.176   0.112   0.970   0.001   0.030
B L  4BJ5A                363    0.088  16.113   0.342   0.970   0.001   0.030
E D  4BJ5A                364    0.553  79.644   0.683   0.923   0.002   0.076
E K  4BJ5A                365    0.437  89.911   1.506   0.938   0.007   0.055
B I  4BJ5A                366    0.047   8.639  -0.154   0.879   0.010   0.111
E R  4BJ5A                367    0.391  89.608   0.372   0.802   0.014   0.185
E N  4BJ5A                368    0.585  85.673  -0.805   0.522   0.016   0.462
E R  4BJ5A                369    0.345  79.097   0.756   0.386   0.097   0.517
B L  4BJ5A                370    0.092  16.772   0.146   0.231   0.330   0.439
E T  4BJ5A                371    0.361  50.098   0.266   0.079   0.592   0.329
B I  4BJ5A                372    0.126  23.347  -0.977   0.079   0.592   0.329
E K  4BJ5A                373    0.525 108.013   0.086   0.022   0.359   0.619
E N  4BJ5A                374    0.567  83.038  -1.364   0.020   0.205   0.775
B Y  4BJ5A                375    0.206  44.022   0.955   0.018   0.088   0.893
E P  4BJ5A                376    0.430  61.003  -0.895   0.018   0.047   0.935
E H  4BJ5A                377    0.370  67.303  -1.018   0.052   0.084   0.864
E S  4BJ5A                378    0.437  51.228  -1.173   0.113   0.087   0.800
B A  4BJ5A                379    0.307  33.853  -1.548   0.118   0.150   0.732
B Y  4BJ5A                380    0.298  63.683  -0.465   0.125   0.227   0.648
E N  4BJ5A                381    0.482  70.506  -0.460   0.125   0.227   0.648
B F  4BJ5A                382    0.170  34.079  -0.430   0.125   0.227   0.648
E K  4BJ5A                383    0.540 111.057   0.803   0.125   0.227   0.648
E K  4BJ5A                384    0.578 118.853   0.180   0.118   0.150   0.732
E N  4BJ5A                385    0.574  84.063  -1.181   0.118   0.150   0.732
E Q  4BJ5A                386    0.481  85.853  -0.277   0.064   0.216   0.721
E R  4BJ5A                387    0.506 115.851   0.357   0.066   0.296   0.638
B L  4BJ5A                388    0.128  23.492   0.783   0.064   0.216   0.721
E P  4BJ5A                389    0.336  47.622  -0.205   0.135   0.317   0.548
B L  4BJ5A                390    0.153  28.069  -0.006   0.268   0.505   0.227
E K  4BJ5A                391    0.501 103.035   0.672   0.268   0.505   0.227
B L  4BJ5A                392    0.135  24.719  -0.079   0.373   0.401   0.226
B T  4BJ5A                393    0.300  41.665  -0.403   0.478   0.309   0.214
E R  4BJ5A                394    0.462 105.890   0.033   0.453   0.248   0.299
E K  4BJ5A                395    0.533 109.679   0.709   0.578   0.229   0.194
B V  4BJ5A                396    0.235  36.166  -0.803   0.428   0.171   0.402
E H  4BJ5A                397    0.480  87.276  -0.841   0.278   0.093   0.628
E D  4BJ5A                398    0.745 107.326  -0.817   0.113   0.043   0.844
E R  4BJ5A                399    0.791 181.208   0.408   0.003   0.003   0.994

Explain the output.  Go back.
```

---
